# The Evolution of Tau Phosphorylation and Interactions

**DOI:** 10.3389/fnagi.2019.00256

**Published:** 2019-09-18

**Authors:** Nataliya I. Trushina, Lidia Bakota, Armen Y. Mulkidjanian, Roland Brandt

**Affiliations:** ^1^Department of Neurobiology, University of Osnabrück, Osnabrück, Germany; ^2^Department of Physics, University of Osnabrück, Osnabrück, Germany; ^3^School of Bioengineering and Bioinformatics, Lomonosov Moscow State University, Moscow, Russia; ^4^A.N. Belozersky Institute of Physico-Chemical Biology, Lomonosov Moscow State University, Moscow, Russia; ^5^Center for Cellular Nanoanalytics, University of Osnabrück, Osnabrück, Germany; ^6^Institute of Cognitive Science, University of Osnabrück, Osnabrück, Germany

**Keywords:** disorder, microtubule-associated protein, phosphorylation, tau, tauopathy

## Abstract

Tau is a neuronal microtubule-associated protein (MAP) that is involved in the regulation of axonal microtubule assembly. However, as a protein with intrinsically disordered regions (IDRs), tau also interacts with many other partners in addition to microtubules. Phosphorylation at selected sites modulates tau’s various intracellular interactions and regulates the properties of IDRs. In Alzheimer’s disease (AD) and other tauopathies, tau exhibits pathologically increased phosphorylation (hyperphosphorylation) at selected sites and aggregates into neurofibrillary tangles (NFTs). By bioinformatics means, we tested the hypothesis that the sequence of tau has changed during the vertebrate evolution in a way that novel interactions developed and also the phosphorylation pattern was affected, which made tau prone to the development of tauopathies. We report that distinct regions of tau show functional specialization in their molecular interactions. We found that tau’s amino-terminal region, which is involved in biological processes related to “membrane organization” and “regulation of apoptosis,” exhibited a strong evolutionary increase in protein disorder providing the basis for the development of novel interactions. We observed that the predicted phosphorylation sites have changed during evolution in a region-specific manner, and in some cases the overall number of phosphorylation sites increased owing to the formation of clusters of phosphorylatable residues. In contrast, disease-specific hyperphosphorylated sites remained highly conserved. The data indicate that novel, non-microtubule related tau interactions developed during evolution and suggest that the biological processes, which are mediated by these interactions, are of pathological relevance. Furthermore, the data indicate that predicted phosphorylation sites in some regions of tau, including a cluster of phosphorylatable residues in the alternatively spliced exon 2, have changed during evolution. In view of the “antagonistic pleiotropy hypothesis” it may be worth to take disease-associated phosphosites with low evolutionary conservation as relevant biomarkers into consideration.

## Introduction

The microtubule-associated protein (MAP) tau is involved in the regulation of neuronal microtubule (MT) assembly. Together with the closely related MAP2, it has arisen in evolution after a duplication of an ancestral gene of ancient cyclostomes (Sündermann et al., 2016). Tau and MAP2 contain a similar C-terminal region (CTR) with a highly conserved microtubule-binding region (MBR), while their amino-terminal sequences vary (Dehmelt and Halpain, 2005). It has been proposed that this variance plays a role in the differential localization and function of these proteins in cells, as tau is enriched in the axon, whereas MAP2 is localized to the somato-dendritic compartment (Weissmann et al., 2009).

Tau is encoded by a single gene, which, in the human genome, is located on chromosome 17q21 (Andreadis et al., 1992). In the human central nervous system (CNS) tau is expressed in several alternatively spliced isoforms, of which the longest isoform is encoded by 11 exons and contains 441 amino acids (aa); three of the exons (exons 2, 3 and 10) are alternatively spliced to generate six different CNS isoforms (Bakota et al., 2017). Tau is also subject to many posttranslational modifications (PTMs) including phosphorylation, O-glycosylation, ubiquitination, methylation, acetylation, sumoylation and proteolytic cleavage, which, together with the different splice variants, generates many proteoforms that may exhibit different localization and functional specialization (Heinisch and Brandt, 2016; Guo et al., 2017). The best-studied PTM of tau is phosphorylation since tau belongs to the major phosphoproteins in the brain.

Tau can aggregate into neurofibrillary tangles (NFTs), which are a hallmark of several neurodegenerative diseases collectively called “tauopathies” (Arendt et al., 2016). The most common tauopathy is Alzheimer’s disease (AD), where intracellular NFTs are joined by the presence of extracellular amyloid plaques containing aggregated amyloid-β (Aβ). Analysis of the histopathological changes in patients with AD has shown that tau inclusions correlate much better with cognitive impairment than amyloid plaques (Nelson et al., 2012) suggesting a major role of changes in tau expression and phosphorylation for disease development. NFTs are composed of tau proteins with increased phosphorylation (“hyperphosphorylation”) in paired helical filaments (PHFs) or straight filaments (Crowther, 1991). Dysregulation of tau splicing resulting in the expression of longer tau isoforms at the expense of shorter ones has been shown to be associated with the development of tauopathies and tau aggregation (Goedert et al., 1998). Indeed, increased expression of longer tau isoforms containing exon 10 without a change in total tau induced pathological changes in human tau-expressing mice including increased tau phosphorylation and a shift towards oligomeric tau (Schoch et al., 2016).

Structurally, tau belongs to the class of intrinsically disordered proteins (IDPs), which are known to interact with many binding partners and are considered to be involved in various signaling and regulation processes (Brandt and Leschik, 2004; Uversky, 2015). As an IDP, tau contains intrinsically disordered regions (IDRs), which lack well-defined, three-dimensional structures at physiological conditions, provide a larger interaction surface area and exhibit conformational flexibility. IDRs differ in amino acid composition from regions with defined secondary structures, which allows predicting the presence of IDRs by bioinformatics means. In particular, IDRs contain higher numbers of hydrophilic amino acids and fewer hydrophobic ones (Dyson and Wright, 2005). They may also have more proline and serine residues, which are considered to be disorder-promoting (Atkins et al., 2015). IDRs also bear diverse PTMs, which, in many cases, were shown to have functional importance (Babu et al., 2011; Bah and Forman-Kay, 2016; Darling and Uversky, 2018). As argued by Darling and Uversky, IDRs appear to be the main targets of PTM-catalyzing enzymes (Darling and Uversky, 2018). The available crystal structures of complexes between protein-modifying enzymes (kinases, phosphatases, deacetylases, methyltransferases, glycosyltransferases) and peptides derived from their protein targets show the same mechanism of interaction where the extended, unfolded peptide fits into a narrow grove of the enzyme (Darling and Uversky, 2018). It appears that even the structured protein regions should transiently unfold to be modified. After the very existence of IDRs was postulated (Dunker et al., 2001), many well-studied sites of PTMs were mapped to IDRs of respective proteins (Darling and Uversky, 2018). Although the functional consequences from PTMs of IDRs are extensively described in recent comprehensive reviews (Bah and Forman-Kay, 2016; Darling and Uversky, 2018), it is not possible to predict the impact of a PTM on a particular interaction, i.e., to say whether the PTM will strengthen the interaction or, in contrast, make it weaker. The reason for this ambiguity is that the mechanism of interaction between IDRs and their protein partners is poorly understood (Darling and Uversky, 2018). In the absence of a general understanding, case-by-case studies appear to be the main option.

**Figure 1:**
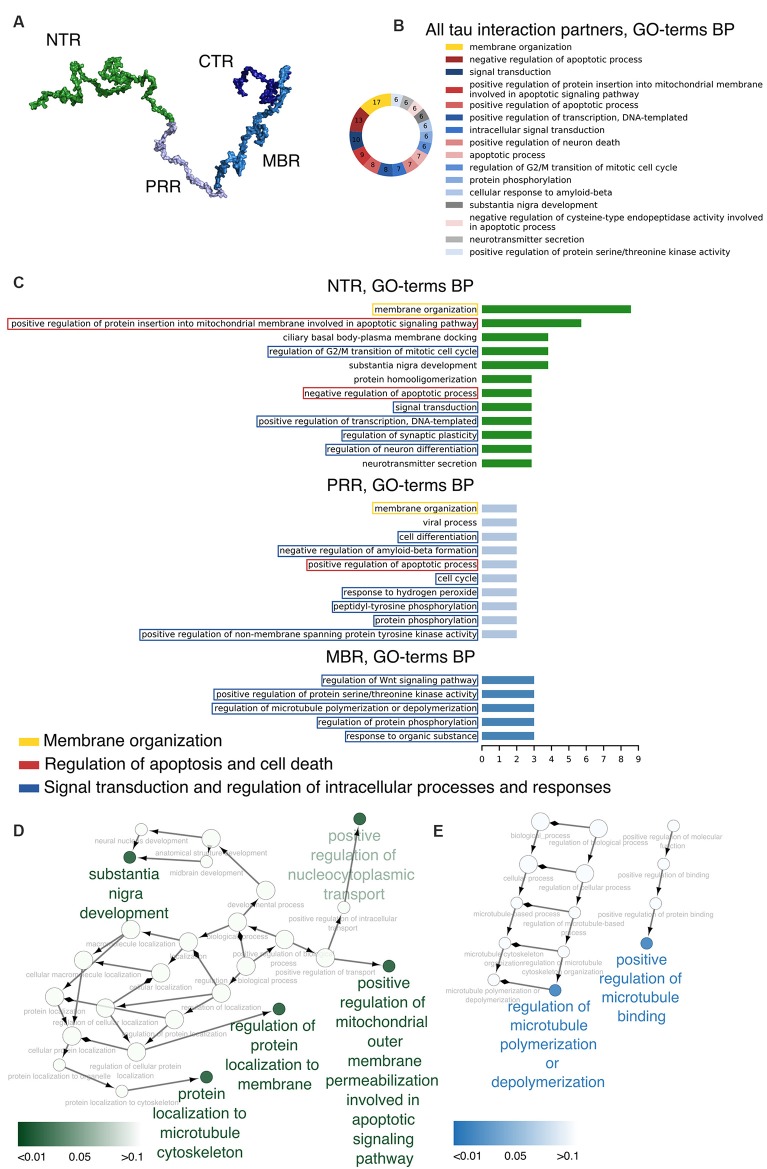
Distinct regions of tau show functional specialization in their molecular interactions. **(A)** A potential 3D structure of tau [441 amino acid (aa) central nervous system (CNS) isoform] generated by the Random Coil Generator (RCG; Jha et al., 2005) is shown. Tau’s region organization was mapped onto the model and regions were color-coded as follows: amino-terminal region (NTR, 1–171)—green; proline-rich region (PRR, 172–243)—light blue; microtubule-binding region (MBR, 244–368)—blue; carboxy-terminal region (CTR, 369–441)—dark blue. **(B)** Most common Gene Ontology (GO)-terms for biological processes (BP) of tau’s interaction partners are shown. **(C)** Bar plots indicate most frequent GO-terms for BP associated with interaction partners of tau, which have been mapped to interact with the NTR, PRR and MBP. Processes associated with membrane organization are shown in yellow boxes, with apoptosis and cell death-related processes in red boxes, and with signaling and complex regulation processes in blue boxes. **(D,E)** GO-term enrichment analysis for genes identified as interaction partners of tau’s NTR **(D)** and MBR **(E)** was performed. Enriched GO-terms for BP are presented in the graph. Enrichment analysis was performed for *Homo sapiens* genes.

Based on sequence and functional properties, four regions of tau can be differentiated (Figure 1A; Bakota et al., 2017). These are the N-terminal projection region (NTR), which protrudes from the MT surface when tau interacts with MTs, the MBR, and the proline-rich region (PRR) and CTR, which flank the MBR amino-terminally and carboxy-terminally, respectively. The carboxy-terminal half of tau containing the MBR and CTR is highly conserved in mammals, birds, reptiles and bony fish and is also present in other members of the MAPT/MAP2/MAP4 family of MAPs. This region contains several repeats of a highly similar motif with a length of 18 amino acids (Niewidok et al., 2016) and encompasses the main microtubule-interacting sites (Kadavath et al., 2015; Kellogg et al., 2018). The NTR and PRR are much less conserved and may represent regions, where compartment-specific, non-microtubule related interactions of tau developed during evolution. These regions may also have contributed to tau’s malfunction during the development of tauopathies, which occur in higher vertebrates. In fact, the NTR and PRR contain IDRs, which may play diverse roles in the modulation and control of the functions of many different binding partners (Uversky, 2015; Trushina et al., 2019). Alternatively spliced exons of tau CNS isoforms code for regions that are located in the NTR (exon 2 and 3) and in the MBR (exon 10); exon 10 codes for an additional microtubule-interacting site (Figure 4B).

**Figure 2:**
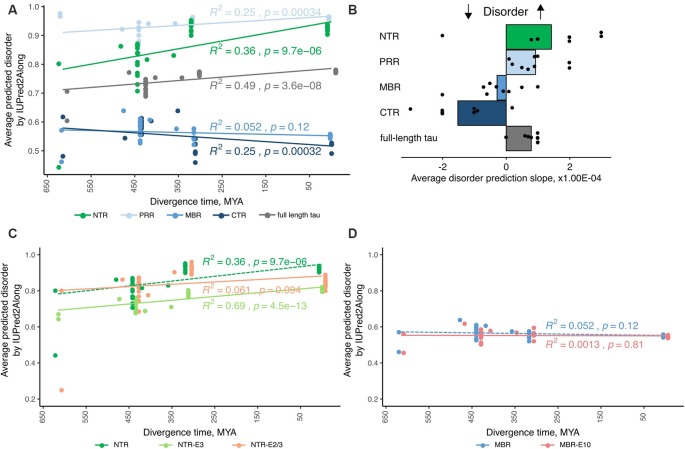
Tau’s amino-terminal, non-microtubule binding region exhibits a strong evolutionary increase in disorder. **(A)** An exemplary plot of disorder prediction by IUPred2A long is shown. Species are grouped by divergence times between their higher taxons and mammals [cyclostomes or jawless fishes—615 million years ago (MYA), cartilaginous fishes—473 MYA, bony fishes—435 MYA, coelacanths—413 MYA, amphibians—352 MYA, reptiles and birds—312 MYA and mammals as a 0 value]. R squared and *p*-value characterizing the linear fit to the data are presented close to each respective fitting line. **(B)** Average values of slopes based on linear regression analysis of predicted disorder values from all selected algorithms for full-length tau and all selected regions (NTR—green, PRR—light blue, MBR—blue and CTR—dark blue) are shown. Black dots represent values for measured slopes for the individual prediction algorithms while bars show average values. Note, that NTR and PRR show an increase in disorder, while the CTR shows a decrease. **(C,D)** Plots of disorder prediction by IUPred2A for NTR lacking exon 2/3 or exon 3 **(C)** and MBR lacking exon 10 **(D)** are shown. Full-length NTR and MBR as also presented in **(A)** are indicated by dashed lines. Species are grouped as described before. R squared and *p*-value characterizing the linear fit to the data are presented close to each respective fitting line.

**Figure 3:**
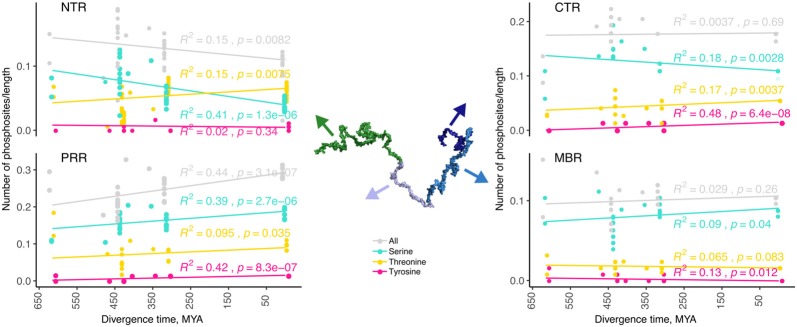
The number of predicted phosphorylation sites changes during evolution in a region-specific manner. Predicted phosphorylation sites for the different regions of tau (NTR, PRR, MBR and CTR) are shown. The numbers of predicted sites were normalized by the length of the analyzed regions. Selected species were grouped by divergence times between mammals and other groups of vertebrates. Coefficients of determination and *p*-values for the linear regression model are presented near the line fits. Fits with *p*-values higher than 0.05 are considered to show no significant relationship between divergence times and normalized numbers of predicted phosphorylation sites. Note, that the PRR is the only region where overall phosphorylation increases during evolution.

**Figure 4:**
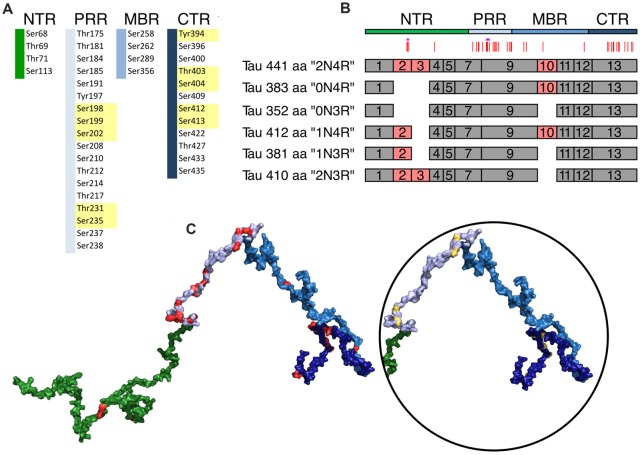
Schematic representation of disease-associated phosphorylation sites in tau. **(A)** The distribution of tau phosphorylation sites as they have been determined by mass spectroscopy of PHF tau from brains of Alzheimer’s disease (AD) patients is shown (based on Hanger et al., 2007). The different tau regions are indicated in green (NTR), gray (PRR), light blue (MBR) and dark blue (CTR). Numbers are based on tau from *Homo sapiens* (441 aa CNS isoform). Major phosphorylation sites, are shown in yellow (based on Morishima-Kawashima et al., 1995). **(B)** Schematic representation of splice isoforms of tau in the CNS. Alternatively, spliced exons are indicated as red boxes. Vertical red lines on top represent the phosphorylation sites listed in **(A)**. The STPT site and the YSSPGS motif are shown by pink and violet horizontal lines, respectively. **(C)** Location of the phosphorylation on one of the potential 3D structures of tau (441 aa isoform) generated by the RCG (Jha et al., 2005) is shown. The sites from **(A)** are indicated in red. In the circle, the positions of the 10 major phosphorylation sites, which are clustered in the PRR and CTR, are indicated.

We have previously provided evidence that evolutionary changes in tubulin-structure proteins, MT-binding proteins and tubulin-sequestering proteins are prominent drivers for the development of increased neuronal complexity (Trushina et al., 2019). We also provided evidence that tau displays an increase in disorder extent during evolution. However, potential consequences with respect to the different functional interactions of tau remained open and changes in sites for PTMs during evolution were not addressed. In this study, we examined published results of tau’s interactions by bioinformatics means to extract information about potential functional specialization of individual regions of tau protein. We determined changes in disorder of individual regions of tau with the help of multiple disorder prediction algorithms and examined changes in the number of predicted phosphorylated residues throughout evolution. In addition, we examined the distribution and conservation of potentially disease-associated tau phosphorylation sites.

## Materials and Methods

### GO-Term Analysis

Uniprot^1^ IDs for some of tau’s interaction partners were retrieved for the respective human proteins; their QuickGO^2^ annotations were searched for Gene Ontology (GO)-terms corresponding to Biological Process (BP). Different cut-off values for the number of proteins associated with most frequent GO-terms were selected (six for all interaction partners regardless of the region, three for NTR and MBR and two for PRR, as it had the lowest number of annotated interaction partners). Data analysis and visualization were performed with Cytoscape 3.7.1 (Shannon et al., 2003), GO-term enrichment was performed using ClueGO plug-in (Shannon et al., 2003; Bindea et al., 2009).

### Modeling Tau 3D Structure

A potential structural model of the longest human CNS tau isoform (441 aa isoform) was generated by the Random Coil Generator (RCG) software (Jha et al., 2005) with side-chain conformation predicted by Scwrl4 with standard parameters (Krivov et al., 2009). The random coil model is frequently used to generate conformational ensembles of IDPs. The structure was represented as a surface; visualization and structure rendering was performed using PyMOL^3^. Tau’s region organization was mapped onto the model with the following color-code: NTR—green, PRR—light blue, MBR—blue and CTR—dark blue. The surface of residues that were shown to be phosphorylated was colored red and major phosphorylation sites were colored yellow.

### Selecting Tau Sequences From Various Species

MAPT sequences were retrieved from Uniprot^1^ and RefSeq Release 93^4^ databases; we also used the sequences that were manually cured for previous work in our laboratory (Sündermann et al., 2016). The best represented higher taxons in our analysis were bony fish (13 sequences), reptiles and birds (2 and 11 sequences, respectively) and mammals (16 sequences). We also included into our analysis sequences from cyclostomes—hagfish (*Eptatretus burgeri*) and lamprey (*Petromyzon marinus*), cartilaginous fish (*Callorhinchus milii*), coelacanth (*Latimeria chalumnae*) and amphibian (*Xenopus tropicalis*). The selected organisms were grouped according to higher taxons (Cyclostomata, Chondrichthyes, Actinopterygii, Coelacanthiformes, Amphibia, Sauropsida, Mammalia) and were color-coded from light yellow to orange in the respective order. The following estimated divergence times between mammals and other groups of vertebrates were obtained from TimeTree^5^: Cyclostomata—615 MYA (million years ago), Chondrichthyes—473, Actinopterygii—435, Coelacanthiformes—413, Amphibia—352, Sauropsida—312 and Mammalia as 0 (Kumar et al., 2017). We included hagfish and lampreys as they are considered to be at the origin of all vertebrates and to have diverged shortly after the separation of cyclostomes (Kuraku and Kuratani, 2006).

### Disorder Prediction

The following disorder prediction algorithms were used to analyze disorder of different tau regions: (1) IUPred2A (long, short); predicts structural disorder based on a biophysical model and optimized for different length disordered regions (Dosztányi et al., 2005). (2) PONDR^®^ (VLXT, VL3-BA and VSL2); mostly neural networks that were trained on different datasets of ordered and disordered proteins to predict disorder for analyzed sequences (Romero et al., 2001; Peng et al., 2006). (3) SLIDER; gives the score resembling how likely a protein has long disorder segment (Peng et al., 2014). (4) Espritz (N—NMR, X—X-ray and D—Disprot); networks that were trained on different datasets, e.g., NMR mobility data, X-ray crystallography of short disorder and Disprot data for long disorder (Walsh et al., 2012).

### Phosphorylation Database Use and Phosphorylation Prediction

We used the following phosphorylation databases and prediction software: (1) PhosphoSitePlus^®^^6^-PTM database containing information on experimentally observed modifications (Hornbeck et al., 2015); (2) NetPhos^7^ (Blom et al., 1999) for prediction of phosphorylation sites.

### Alignment and Disorder Mapping

Alignment of selected sequences was performed with Tcoffee (Notredame et al., 2000) and further edited manually. To map the predicted disorder scores onto the alignment the predictions from IUPred2A were used.

### Statistical Analysis

Two-sided Mann–Whitney U test was used to analyze the differences between most represented groups of vertebrates’ predicted disorder and phosphorylation. Significance levels were defined as follows: *p ≤ 0.05, **p ≤ 0.01, ***p ≤ 0.001, ****p ≤ 0.0001. Linear regression analysis was used to evaluate the changes throughout tau sequences of all selected species.

## Results

The longest isoform of tau (often called “big tau”; Goedert et al., 1992) is produced specifically in the peripheral nervous system (PNS) and is encoded by 14 exons generating a protein with an apparent molecular mass of about 110 kDa. However, due to the involvement of tau in pathologies of the CNS, most functional and structural studies concentrated on tau isoforms expressed from the MAPT gene in CNS neurons; thus, we will restrict our analysis to the longest CNS tau isoform containing 441 amino acids (aa) in humans, which is encoded by 11 exons (Andreadis et al., 1992) including the three exons, which are alternatively spliced in the CNS (exon 2, 3 and 10; Figure 4B).

### Distinct Regions of Tau Show Functional Specialization in Their Molecular Interactions

To determine potential functionally relevant interactions of different regions of tau protein with cellular binding partners, we first performed a literature search for cellular components, which have been reported to interact with tau. All together the search revealed about 60 different interaction partners. About one-third of the interactions were reported to be sensitive to phosphorylation. Table 1.1 shows a summary of the results for tau binding partners, whose interactions have been mapped to specific regions of tau protein. Table 1.2 displays additional partners, whose interaction sites have not been mapped. Genes and Uniprot IDs for the respective human genes coding for the interaction partners are shown in Supplementary Table S1.

**Table 1.1:** Tau binding partners, whose interactions have been mapped to specific regions of tau protein.

Protein	Gene name (*H. sapiens*)	Selected references	Potential physiological function
**N-terminal projection region (NTR)**			
End-binding proteins	MAPRE1, MAPRE3	Sayas et al. (2019)	regulation of microtubule dynamics
Annexin A2	ANXA2	Gauthier-Kemper et al. (2018)	contribution to tau’s axonal localization
Annexin A6	ANXA6	Gauthier-Kemper et al. (2018)	contribution to tau’s axonal localization
Annexin A5	ANXA5	Stefanoska et al. (2018)
14–3–3β, 14–3–3β	YWHAB, YWHAH	Stefanoska et al. (2018)	modulation of cell signaling
Synapsin-1	SYN1	Stefanoska et al. (2018)	modulation of transmitter release
Synaptotagmin-1	SYT1	Stefanoska et al. (2018)	modulation of transmitter release
14–3–3ε, γ, ζ, and σ	YWHAE, YWHAG, YWHAZ, SFN	Tugaeva et al. (2017)	modulation of ell signaling
Synaptic vesicles	VAMP2	Zhou et al. (2017)	modulation of transmitter release
Prion protein (PrP)	PRNP	Wang et al. (2008)
Dynactin complex	DCTN1	Magnani et al. (2007)	modulation of organelle transport
Heparin	NA	Sibille et al. (2006)
Glycogen synthase kinase-3β	GSK3B	Sun et al. (2002)	modulation of axonal phosphorylation
Membrane cortex	ACTB	Brandt et al. (1995) and Maas et al. (2000)	contribution to tau’s axonal localization
**Proline-rich region (PRR)**			
Bridging integrator-1 (BIN1)	BIN1	Malki et al. (2017) and Sottejeau et al. (2015)
14–3–3σ	SFN	Joo et al. (2015)	modulation of cell signaling
DNA	NA	Qi et al. (2015)	transcriptional regulation
Protein phosphatase PP2A/Bα	PPP2R2A	Sontag et al. (2012)	modulation of axonal phosphorylation
Src-family non-receptor tyrosine kinases fyn	FYN	Lee et al. (1998) and Usardi et al. (2011)	modulation of cell signaling
Heparin	NA	Sibille et al. (2006)
Peptidyl prolyl cis/trans-isomerase Pin1	PIN1	Smet et al. (2004)
Src-family non-receptor tyrosine kinases src	SRC	Lee et al. (1998)	modulation of cell signaling
**Microtubule-binding region (MBR)**
Heat shock cognate 71 kDa protein (Hsc70)	HSPA8	Taylor et al. (2018) and Sarkar et al. (2008)
Small heat shock protein HspB1/heat shock protein 27 (Hsp27)	HSPB1	Freilich et al. (2018)
End-binding proteins (EBs)	MAPRE1, MAPRE3	Ramirez-Rios et al. (2016)	regulation of microtubule dynamics
14–3–3σ	SFN	Joo et al. (2015)	modulation of cell signaling
DNA	NA	Qi et al. (2015)	transcriptional regulation
Amyloid β (Aβ)	APP	Pérez et al. (2004) and Manczak and Reddy (2013)
Protein disulfide isomerase (PDI)	P4HB	Xu et al. (2013)	
Protein phosphatase PP2A/Bα	PPP2CA	Sontag et al. (2012)	modulation of axonal phosphorylation
Heparin	NA	Zhu et al. (2010) and Sibille et al. (2006)
Histone deacetylase 6	HDAC6	Ding et al. (2008)	transcriptional regulation
Prion protein (PrP)	PRNP	Wang et al. (2008)
Phosphatase 2A (PP2A) isoform ABalphaC	PPP2CA	Sontag et al. (1999) and Eidenmuller et al. (2000)	modulation of axonal phosphorylation
Tau (tau-tau interaction)	MAPT	Pérez et al. (1996)
Actin filaments	ACTB	Correas et al. (1990), Moraga et al. (1993) and Selden and Pollard (1986)	regulation of cytoskeletal interactions
Microtubules	NA	Butner and Kirschner (1991), Lee et al. (1989) and Gustke et al. (1992)	regulation of microtubule dynamics
Calmodulin	CALM1	Padilla et al. (1990)	modulation of cell signaling
**C-terminal region (CTR)**			
Microtubules	NA	Kadavath et al. (2015)	regulation of microtubule dynamics
Heparin	NA	Sibille et al. (2006)

*Interactions, which have been reported to be sensitive to phosphorylation, are indicated in red color*.

**Table 1.2:** Additional binding partners, whose interactions have not been mapped to tau specific regions.

Protein	Gene name (*H. sapiens*)	Selected references	Potential physiological function
**Additional interactions (not mapped)**
α-synuclein	SNCA	Yan et al. (2018) and Esposito et al. (2007)	modulation of transmitter release
DEAD box RNA helicase DDX6	DDX	Chauderlier et al. (2018)
Heat shock protein 90 (Hsp90)	HSP90AB1	Radli and Rüdiger (2018)
Voltage dependent anion channel (VDAC) proteins	VDAC1	Magri and Messina (2017)
Transferrin	TF	Jahshan et al. (2016)
Ferritin	FTH1	Jahshan et al. (2016)
FK506-binding protein 4 (FKBP52)	FKBP4	Kamah et al. (2016)
RNA-Binding Protein TIA1	TIA1	Vanderweyde et al. (2016)	modification of stress response
Death-associated protein kinase 1 (DAPK1)	DAPK1	Pei et al. (2015)
Leucine-rich repeat kinase 2 (LRRK2)	LRRK2	Shanley et al. (2015)
Clusterin (apolipoprotein J)	CLU	Zhou et al. (2014)
14–3–3zeta	YWHAZ	Qureshi et al. (2013), Sluchanko et al. (2011) and Sluchanko et al. (2009)
Dynamin-related protein (Drp1)	DNM1L	Manczak and Reddy (2012)
Phospholipid membranes	NA	Künze et al. (2012)
Synaptic proteins	SYN1, SYT1, VAMP2	Mondragón-Rodríguez et al. (2012)	modulation of transmitter release
C-Jun N-terminal kinase-interacting protein 1 (JIP1)	MAPK8IP1	Ittner et al. (2009)
Golgi membranes	NA	Farah et al. (2006)
Fe65 protein	APBB1	Barbato et al. (2005)
Rb binding protein Che-1	AATF	Barbato et al. (2003)
Alu-derived domain	NA	Hoenicka et al. (2002)
S100b	S100B	Baudier and Cole (1988) and Yu and Fraser (2001)
Cdc2-like protein kinase	CDK2	Sobue et al. (2000)	modulation of axonal phosphorylation
Presenilin 1	PSEN1	Takashima et al. (1998)
Apolipoprotein E	APOE	Richey et al. (1995) and Fleming et al. (1996)
Phosphatidylinositol	NA	Surridge and Burns (1994)
Spectrin	SPTAN1	Carlier et al. (1984)	regulation of cytoskeletal interactions

*Interactions, which have been reported to be sensitive to phosphorylation, are indicated in red color*.

The data indicate that tau interacts with a variety of different proteins and cellular components, which appear to be different for the NTR, the PPR and the MBR, respectively. None of the interaction partners was specific for the CTR. Interestingly, many interaction partners of tau appeared to be structurally unrelated to each other, which may be due to tau’s intrinsic disorder properties that is associated with the presence of multiple interactions with relatively low affinity (Babu et al., 2011). To determine potentially common functional features of the interaction partners, we analyzed GO-terms associated with biological processes (BP) for the interacting partners (Figure 1B). Most GO-terms of the interaction partners (Tables 1.1, 1.2; Supplementary Table S1) were related to “membrane organization,” “regulation of apoptotic processes” and “signal transduction.” When the most frequent GO-terms were grouped for the individual tau regions, we observed that “membrane organization” and “regulation of apoptotic processes” were among the GO-terms, which were specific for the amino-terminal part of tau (NTR and PRR), while all common GO-terms for the MBR were “signal transduction and regulation of intracellular processes and responses” (Figure 1C).

GO-term enrichment analysis for genes identified as interaction partners of tau’s NTR and MBR, respectively, confirmed the functional specialization of these two regions (Figures 1D,E). We observed a strong enrichment for “regulation of protein localization to membrane” and “involvement in apoptotic signaling pathway” for the NTR, while the MBR was particularly specialized for “regulation of MT polymerization.”

Thus, the data indicate that the highly conserved MBR on one side and the much less conserved N-terminal region on the other side show specific functional specialization. The data also suggest that in particular tau’s potential functions with respect to membrane organization and regulation of apoptotic processes are mediated *via* the NTR and PRR. Until to date, no interaction partners have been identified, which specifically interact with sequences encoded by the three alternatively spliced exons in CNS tau (exons 2, 3 and 10). However, experiments using recombinant tau fragments have provided evidence that segments encoded by exons 2 and 10 promoted tau aggregation, whereas the segment encoded by exon 3 depressed it (Zhong et al., 2012). Thus, sequences that are encoded by exons 2, 3 and 10 may modulate tau-tau interaction rather than contributing additional interactions. This is in line with data showing that an increase in isoforms containing exon 10 leads to the formation of abnormal tau filaments in patients with tauopathies (Spillantini et al., 1998) and that carriers of the MAPT H2 haplotype, which express more tau containing exons 2/3 than H1 carriers, appear to be more protected from neurodegeneration (Caffrey et al., 2008). It has also been reported that the peptide NAP (davunetide, CP201), which has been tested in clinical trials for the treatment of tauopathies, preferentially interacts with tau isoforms lacking exon 10 suggesting that some interactions may also be specific for shorter tau isoforms (Ivashko-Pachima et al., 2019).

### Tau’s Amino-Terminal, Non-microtubule Binding Region Exhibits a Strong Evolutionary Increase in Disorder

Previously, we reported a tendency of increased disorder of tau protein structure during evolution, a feature, which tau shared with MAP6 protein, which is involved in stabilization of axonal microtubules (Tortosa et al., 2017). In contrast, we did not observe a similar increase in disorder in the structure of the somatodendritic MAP2 protein (Trushina et al., 2019). Since IDRs provide a larger interaction surface area and are known to be hubs for cellular interactions, different tau regions may differ in their extent of disorder. Furthermore, evolutionary changes in the extent of disorder of particular regions of tau could indicate, which type of interactions may have evolved during evolutionary development.

IDRs are characterized by the presence of low sequence complexity and amino acid compositional bias (Dyson and Wright, 2005; Atkins et al., 2015), which allows predicting the presence of IDRs by bioinformatics means. To determine the level of disorder within the tau protein and potential changes during evolution, we employed disorder predicting algorithms to determine disorder scores for tau sequences of selected species. Since all of the individual algorithms have constraints because the predictors were trained on different training sets and take into account certain factors while neglecting others, we decided to employ a set of various prediction algorithms and determine average scores.

The species were grouped by divergence times between mammals and other taxonomy groups of vertebrates (cyclostomes or jawless fishes—615 MYA, cartilaginous fishes—473 MYA, bony fishes—435 MYA, coelacanths—413 MYA, amphibians—352 MYA, reptiles and birds—312 MYA and mammals as a 0 value) and the relation of predicted disorder values and divergence times were analyzed with linear regression model. An example of a respective representation of the change in the level of disorder of different tau regions through vertebrate evolution with one disorder predicting algorithm is shown in Figure 2A. To increase the reliability of the disorder prediction, we determined the average disorder prediction slope for full-length tau and the different regions from nine different disorder predicting algorithms (Figure 2B; see “Materials and Methods” section). The disorder extent of full-length tau increased during evolution as judged from most prediction programs (Figure 2B; the prediction of the individual algorithms are shown in Supplementary Figure S1). The increase in protein disorder during vertebrate evolution was highest in the NTR followed by the PRR. In contrast, the MBR and CTR showed a negative trend. In addition, we determined the evolutionary changes in disorder for NTR lacking the alternatively spliced exons 2/3 and 3 (Figure 2C) and for MBR lacking exon 10 (Figure 2D). Interestingly, no significant increase in protein disorder of the NTR was observed in sequence lacking exon 2/3, while lack of exon 3 alone did not change the evolutionary increase in the extent of disorder of the NTR. A lack of exon 10 did not lead to any change.

Thus, the data indicate that in particular tau’s amino-terminal region (NTR and PRR) with a noticeable contribution of the alternatively spliced exon 2 exhibits a strong evolutionary increase in disorder, which suggests that novel interactions in addition to tau’s microtubule-related activities developed during evolution. As discussed before, this may, in particular, involve biological processes related to “membrane organization” and “regulation of apoptotic processes” which are both mediated by this region.

### The Number of Predicted Phosphorylation Sites Changes During Evolution in a Region-Specific Manner and Evolves in the NTR and PRR in an Opposite Manner

Phosphorylation is a common PTM, which regulates the function of many proteins and is also thought to modulate the conformational flexibility and interactions of IDRs. Tau is subject to many phosphorylation events and changes in the pattern, the stoichiometry and the dynamics of phosphorylation have been associated with functional changes of tau during physiological and pathological conditions.

**Figure 5:**
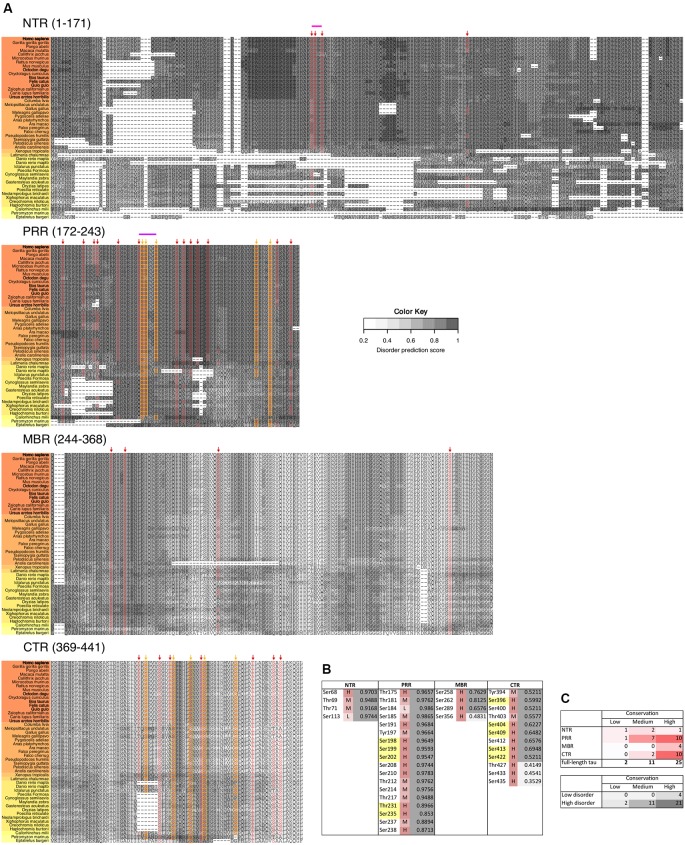
Disease-associated tau hyperphosphorylation sites are highly conserved while many phosphosites in the NTR and PRR exhibit a lower degree of evolutionary conservation. **(A)** Comparison of tau sequences from 47 different species is shown. The selected organisms are grouped according to higher taxons (Cyclostomata, Actinopterygii, Coelacanthiformes, Amphibia, Sauropsida, Mammalia) and are color-coded from yellow (lower vertebrates) to orange (higher vertebrates). The extent of disorder (as predicted by IUPred2A) is indicated by the gray value according to the color key. The position of disease-associated phosphorylation sites as they have been shown in Figure 4 are indicated by red arrows, major phosphorylation sites by yellow arrows. The STPT site and the YSSPGS motif in the human sequence, which show an increase in the number of clustered phosphorylatable residues is shown by pink and violet lines, respectively. **(B)** Level of conservation of the disease-associated phosphorylation sites is shown (H: high conservation—present in >66% of species; M: medium conservation—present in 33%–66% of species; L: low conservation—present <33% of species). The extent of disorder (as predicted by IUPred2A) is also shown. Light gray indicates disorder <0.5, dark gray >0.5. **(C)** Summary representation of the level of conservation of the phosphorylation sites with respect to the region of tau (top), and the extent of disorder (bottom).

Phosphorylation occurs at three different residues, serine, threonine and tyrosine. To determine potential changes in phosphorylation during evolution, we used the NetPhos3 phosphorylation prediction server to obtain numbers of sites that could be phosphorylated with high confidence. After normalization to the number of residues we analyzed the changes in overall phosphosite number and the numbers of serine, threonine and tyrosine phosphosites from different tau regions throughout evolution (Figure 3). The PRR was the only region where overall phosphorylation sites increased, which was due to increased number of serine and threonine phosphorylation sites with divergence time. In contrast, the NTR showed a strong decrease in serine phosphosites, while threonine phosphosites increased. No major changes were observed in the MBR and CTR. Comparison between fish, birds and mammals also revealed a steady increase in overall phosphorylation sites in the PRR, which was mainly due to an increased number of threonine residues and a decrease of serine phosphorylation sites in the NTR (Supplementary Figure S3). In many cases, the overall phosphorylation sites increased owing to the formation of clusters of phosphorylatable residues. Specifically, the STPT site in human tau corresponds to a single potential phosphosite in *Xenopus tropicalis* and simpler organisms (Figures 4B, 5A, pink line). Interestingly, the STPT site is located in the alternatively spliced exon 2. Since inclusion of exon 2 has been shown to promote tau aggregation (Zhong et al., 2012), a change in the phosphorylation pattern within this region may affect tau-tau binding and thus be of pathological relevance. A similar increase in the number of clustered phosphorylatable residues in the course of evolution is seen in the case of the human YSSPGS motif in the PRR (Figures 4B, 5A, violet line).

The data indicates that changes in the number of phosphorylation sites during evolution occurred mainly in the N-terminal part of tau (NTR and PRR), while the number of phosphorylatable residues in the C-terminus (MBR and CTR) remained unaltered. This is in line with the observation that the C-terminus, in general, is evolutionary much more conserved. Interestingly, the NTR and PRR showed opposite changes. While the number of phosphorylation sites in the NTR decreased, which was mostly due to decreased number of serine residues, the number of phosphosites in the PRR showed a strong increase with divergence time.

### Disease-Associated Tau Hyperphosphorylation Sites Are Highly Conserved While Many Phosphosites in the NTR and PRR Exhibit a Lower Degree of Evolutionary Conservation

Pathological changes in tau phosphorylation are thought to induce tau-mediated toxicity and affect tau aggregation (Fath et al., 2002; Noble et al., 2003; Bakota and Brandt, 2016). In most studies, changes in tau phosphorylation in different species have been studied using phosphorylation-sensitive antibodies such as the AT8 antibody, which detects phosphorylated sites at Ser202/Thr205 in the PRR, or the PHF1 antibody, which is specific for a phosphorylated epitope in the CTR (Ser396/Ser404). To avoid bias, which is caused by the availability of antibodies against certain phosphosites but not others and a potential influence of additional phosphorylation events in antibody reactivity (e.g., pSer208 for the reactivity of the AT8 antibody; Malia et al., 2016) or crossreactivity to other epitopes (e.g., cross-reactivity of AT8 to two doubly phosphorylated motifs containing either serines 199 and 202 or serines 205 and 208 of the human tau sequence; Porzig et al., 2007), we restricted our analysis to reports, which have employed mass spectrometry to systematically determine the pattern of phosphorylation.

In tau from PHFs, which have been isolated from patients with AD, a total of 38 phosphorylated sites have been identified by mass spectrometry (Hanger et al., 2007). Ten of the sites represent major phosphorylation sites and may, therefore, qualify as being “hyperphosphorylated” (Morishima-Kawashima et al., 1995). Figure 4 shows the distribution of these sites with respect to the four different regions of tau protein. The majority of the sites and all of the 10 major phosphorylation sites (Figure 4A, yellow boxes) are concentrated in the PRR and the CTR, which flank the MBR on both sides. Additional sites, which are phosphorylated to a lower extent, are also present in the other regions. One can see that in many cases the disease-associated phosphosites shown in Figures 4B, 5A belong to the aforementioned clusters of phosphorylatable residues.

To determine, whether the presence of the phosphosites changed during evolution, we determined the level of conservation of the respective phosphosites by comparing tau sequences across species (Figure 5A). We found that all of the 10 major phosphorylation sites were highly (>66%) conserved across species (Figure 5B). Two sites (Ser113 in the NTR and Ser184 in the PRR) showed a particularly low degree of conservation. In many species, Ser113 was replaced by asparagine and Ser184 by glycine or alanine, i.e., nonpolar amino acids.

The data suggest that most disease-associated phosphorylation events are concentrated in two regions, which flank the MBR on both sites. Also, all of the major phosphorylation sites (hyperphosphorylated sites) are present in these two regions. In general, the disease-associated phosphorylation sites with low conservation are present in the NTR and PRR. All phosphorylation sites in regions of low disorder are highly conserved, whereas about 40% of phosphorylation sites in high disorder regions displayed lower conservation (Figure 5C), which is in line with the observation that IDRs changed during evolution.

### Evolutionary Changes of Specific Tau Phosphorylation Sites of Pathological Relevance

**Figure 6:**
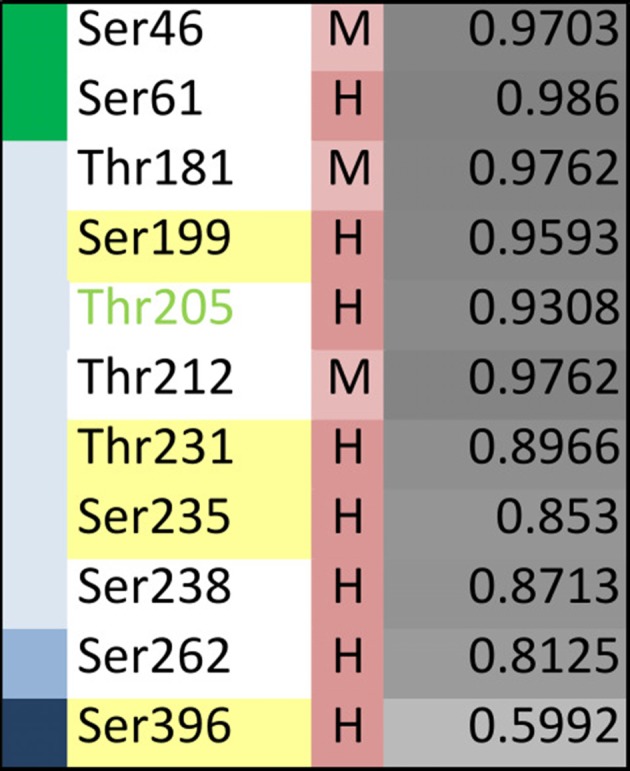
Evolutionary changes of specific tau phosphorylation sites of pathological relevance. Summary representation showing phosphorylation sites that have been reported to be significantly enriched in cerebrospinal fluid (CSF) from AD patients compared to controls (based on Russell et al., 2017). The different tau regions are indicated in green (NTR), gray (PRR), light blue (MBR) and dark blue (CTR). The level of conservation is indicated as described in the legend of Figure 5. The extent of disorder is color-coded from light to dark grey with darker colors showing higher disorder. Thr205 is shown in light-green as its phosphorylation has been shown to decrease Aβ-induced toxicity (Ittner et al., 2016).

Specific phosphosites of tau may be of particular importance during disease development and may be different from those, which have been identified in tau isolated from PHFs. Recent profiling of phosphorylated tau peptides in cerebrospinal fluid (CSF) by mass spectrometry has revealed 11 sites that are increased by at least 40% in the CSF of patients with AD compared to controls (Russell et al., 2017). A summary of the sites together with their localization in different tau regions and the level of conservation is shown in Figure 6. One of the sites is Thr181, an established core biomarker for AD in CSF (Vanmechelen et al., 2000), which has also been detected in PHF tau. Thr181 is one of three sites, which shows only a moderate conservation during evolution. Another site of potential interest is Thr205; phosphorylation of Thr205 has been shown to decrease Aβ-induced toxicity (Ittner et al., 2016) and is therefore the only phosphorylation site, which has been reported to have a protective rather than toxicity-promoting effect. This site is highly conserved but has not yet been identified in tau from PHFs. It should also be noted that two of the CSF-sites are located in the NTR and none of them had been identified in PHFs.

The data show that only partial overlap exists between phosphorylated sites that have been identified in PHF tau and in CSF samples from AD patients. With respect to the identification of phosphorylation sites that may have made tau prone for the development of tauopathies, it may be informative to further analyze those sites that are present in regions which changed during evolution and may have developed novel functions (i.e., NTR and PRR) and which exhibit moderate conservation.

## Discussion

We tested the hypothesis that the sequence of tau has changed during vertebrate evolution in a way that novel interactions developed. We also tested the hypothesis that tau phosphorylation may have been affected by evolutionary changes, which made tau prone for the development of tauopathies. We show that: (1) distinct regions of tau show functional specialization in their molecular interactions; (2) tau’s amino-terminal, non-microtubule binding region exhibits a strong evolutionary increase in protein disorder; and (3) the number of predicted phosphorylation sites changes during evolution in a region-specific manner, while disease-specific hyperphosphorylated sites are highly conserved.

Our data indicate that the highly conserved MBR on one side and the much less conserved N-terminal region on the other side show specific functional specialization. Since the MT-associated proteins tau, MAP2 and MAP4 contain a similar CTR with multiple highly conserved microtubule-binding repeats, tau’s N-terminal region may provide the tau-specific interactions, which may also be involved in its unique subcellular localization with an enrichment in the axonal compartment, which occurs early during neuronal development (Kempf et al., 1996). In fact, GO-terms associated with biological processes for the interaction partners of tau showed a strong enrichment for “regulation of protein localization to membranes” for the NTR, which was clearly different from the MBR, which was mostly associated with “regulation of microtubule polymerization.” Thus, the data suggest that the involvement of tau’s N-terminus in membrane organization is responsible for tau’s axonal enrichment. This is an agreement with previous results that overexpression of tau’s extreme N-terminus interferes with the axonal enrichment of endogenous tau protein in primary neurons (Gauthier-Kemper et al., 2018). Since the MAPT and MAP2 genes are the result of a gene duplication event at the dawn of the vertebrates (Sündermann et al., 2016) it is also likely that the functions related to tau’s localization to the membrane were responsible for the compartment-specific separation of tau and MAPs in the axonal (tau) and somatodendritic (MAP2) compartment.

Interestingly, several interaction partners of the NTR and PRR were also associated with regulation of apoptotic processes, while this was not the case for the MBR and CTR. Current data with respect to an involvement of tau in neuronal survival and regulation of apoptosis are contradictory. On one side it has been reported that overexpression of tau renders cells more resistant to apoptosis, which has been induced by pro-apoptotic factors (Li et al., 2007; Wang et al., 2010). Similarly, accumulation of highly phosphorylated tau, specifically in the cones of primates, has shown to make these cells resilient to apoptosis (Aboelnour et al., 2017). On the other side, tau expression has been shown to be essential for stress-induced brain pathology (Lopes et al., 2016) and reduction of tau ameliorated Aβ induced deficits in a mouse model of AD (Roberson et al., 2007). In one study, anti-apoptotic activity of tau has been located in tau’s amino-terminal region (residues 1–230; Amadoro et al., 2004). In fact, GO-term analysis for biological processes has also identified “negative regulation of apoptotic processes” in the NTR and “positive regulation of apoptotic processes” in the adjacent PRR, which may indicate that different regions of tau may have antagonistic effects with respect to the regulation of neurodegenerative processes, which might be differentially regulated by region-specific phosphorylation.

Tau’s NTR and PRR exhibited an evolutionary increase in disorder, which was particularly strong for the NTR. In contrast, the MBR and CTR exhibited no increase or even a decrease in disorder. Many IDPs and proteins with a substantial extent of IDRs have multiple interaction partners (Uversky, 2015). Thus, an increase in IDRs during evolution may provide a mechanism for increased binding promiscuity and improved ability to adapt to changes in the environment. This suggests that in particular tau’s potential functions with respect to membrane organization and regulation of apoptotic processes, which are both predominantly mediated *via* the NTR, were beneficial for the development of a complex nervous system, which is paralleled by an increased ability of the microtubule system for regulated interactions (Trushina et al., 2019). The involvement of IDPs such as tau in neurodegenerative diseases of mammals could then be interpreted in the context of the “antagonistic pleiotropy hypothesis,” as it was first proposed by Williams (1957). In such a view, increased disorder of tau could be beneficial for the organism’s fitness by providing increased ability for regulated interactions, while it would be detrimental at higher age due to increased propensity for miss-regulation and aggregation.

Intrinsically disordered proteins are thought to develop spread wide structures, which are maintained by networks of weak bonds that become disrupted at higher temperature (Uversky, 2009; Langridge et al., 2014); thus, the body temperature of the respective organisms may influence to what extent disordered regions developed during evolution. Therefore, we determined the changes of the extent of disorder between the tau proteins of poikilotherms (e.g., fishes), which operate at lower temperatures than those of homeotherms (e.g., mammals). As a further comparison we included birds, which belong to the homeotherms but generally operate at higher temperature levels (~41°C) than mammals (Prinzinger et al., 1991). Indeed we observed an increase in disorder as judged from most prediction programs for full-length tau and the NTR from fish to birds but a decrease from birds to mammals (Supplementary Figures S2A,B). For the PRR, we observed a continuous increase from fish to birds to mammals. The results could suggest that the evolutionary increase in disorder for the NTR is influenced by the different body temperature of the organisms in a way that the predicted disorder is highest for species with the highest body temperature. It should also be noted that there are mammalian species, which do change their body temperature at certain seasons. These are the hibernating animals, which show an activity and metabolic depression. These animals regulate this process also by hibernation-state dependent tau phosphorylation in various brain regions. Interestingly, the phosphorylation sites that are undergoing changes in phosphorylation extent and kinetics are overlapping with sites that are stoichiometrically highly phosphorylated during tauopathies (Stieler et al., 2011). This may provide an additional way of modulating the disordered state of tau protein.

We observed that the number of predicted phosphorylation sites changes during evolution in a region-specific manner. Again, changes were most prominent in the amino-terminal part of tau (NTR and PRR). Interestingly, however, the NTR and PRR showed opposite changes in the number of predicted phosphorylation sites during evolution. While the number of predicted phosphorylation sites in the NTR decreased, the PRR showed a strong increase with divergence time and also when fish, birds and mammals were compared. Thus, the data suggest that the propensity of regulation by phosphorylation evolved in an opposite manner in the PRR and the NTR. While it increased in the PRR, it decreased in the NTR. This could also be of relevance for the regulation of apoptotic processes, where the PRR and NTR had an antagonistic effect, which might be differentially regulated by region-specific phosphorylation. Of note, changes in tau phosphorylation during evolution can also be caused by changes in the number and regulation of kinases and phosphatases. Eukaryotic protein kinases regulate almost every biological process and have evolved as dynamic molecular switches (Taylor et al., 2019). However, due to the presence of several subunits in many kinases, which provide high diversity in function and specificity, general statements about the alteration of the activity of kinases towards specific target proteins during evolution are difficult to draw. Attempts are therefore mainly focusing on the analysis of the catalytic subunits. With respect to one of the best-studied kinase, cAMP-dependent protein kinase (PKA) the catalytic subunit isoforms *PRKACA* and *PRKACB* are highly conserved paralogous genes, which result from a gene duplication event around the evolution of the first vertebrate species (Søberg et al., 2013); this is approximately at the same time, when tau has arisen after a duplication of an ancestral gene of ancient cyclostomes (Sündermann et al., 2016). PKA is of particular interest because it modulates GSK3β- and cdk5-catalyzed phosphorylation of tau (Liu et al., 2006). On the other hand, the cyclin-dependent kinases and their regulatory cyclin proteins were evolving much earlier at the level of metazoans, which also indicates their involvement in a much more conserved function, like cell cycle regulation (Cao et al., 2014). Phosphatases are often also composed of several subunits. Furthermore, some need adaptor proteins, which provide phosphatase specificity and pathway insulation in signaling networks (Rowland et al., 2015). Therefore, it is also difficult to follow evolutionary trends that would affect one specific target protein.

The data indicated that most disease-associated phosphorylation events are concentrated in two regions, which flank the MBR on both sites. Also, all of the major phosphorylation sites (“hyperphosphorylated” sites) were present in these two regions. The sequence comparison revealed that all of the hyperphosphorylated sites are highly conserved and thus have the potential to be phosphorylated in most species. Comparison of the occurrence of NFTs in various animal species indicates that certain species such as degu (*Octodon degu*), and domestic cat *(Felis catus)* develop NFTs, while closely related species such as rat (*Rattus norvegicus*) or the domestic dog *(Canis lupus familiaris)* do not (Youssef et al., 2016; Figure 5A). Thus, it appears that the simple presence of these sites cannot explain why NFTs develop in certain species but not in others. With respect to the development of potentially new mechanisms of regulation, which may also become mis-regulated during pathological processes, it may, therefore, be worth to also take phosphosites with lower evolutionary conservation and later development during evolution as relevant biomarkers into consideration. In this context it is interesting to note that tau phosphorylated at the moderately conserved threonine residue 181 (pThr181) is an established core biomarker for AD (Vanmechelen et al., 2000), which shows a significant increase in phosphorylation in the CSF of patients with AD compared to controls (Russell et al., 2017). Furthermore, it has also been shown that pThr181 has a potential to serve as a predictor during differential diagnosis while it showed a significant increase in phosphorylation in the CSF of patients with mild cognitive impairment (MCI), whose diseases progresses to AD, compared to patients with MCI showing stable cognitive function. It has also been shown that several non-AD type dementias exhibit a significant lower phosphorylation level at Thr181 compared to CSF samples from AD patients (Kang et al., 2013).

Noteworthy, some of the highly conserved phosphorylation sites are not having exclusively a disease relation but are also highly phosphorylated during development as it was shown in the hippocampus of kittens where phosphorylation of tau at Ser202/Thr205 disappeared from axons above 1 month of age (Riederer et al., 2001) or human hippocampal biopsy samples where the number of Ser199 immunoreactive neuronal cells were counted high at very young age and were steeply declining during aging in healthy individuals (Maurage et al., 2001).

The observed increase in the number of clustered phosphorylatable residues in the course of evolution (Figure 5A) strongly resembles duplications of functionally important aspartate residues in case of the apoptotic protease activating factor 1 (Apaf-1). In the intrinsic apoptotic cascade, cytochrome c, when released from damaged mitochondria, binds between the tryptophan (W) and aspartate (D)-rich WD-8 and WD-7 domains of Apaf-1. This binding causes a major conformational change that eventually could lead to the assembly of apoptosome. Molecular modeling predicted that cytochrome c binds owing to the interaction of its lysine residues with conserved Asp residues of Apaf-1 (Shalaeva et al., 2015). The recently resolved apoptosome structure confirmed the involvement of conserved Asp residues in cytochrome c binding (Zhou et al., 2015). In four of such conserved sites, a single Asp residue appears to be replaced by a Asp-Asp dimer upon transition from primitive organisms to Chordates (Shalaeva et al., 2015).

In case of Apaf-1, it was speculated that an Asp-Asp dimer could provide stronger hydrogen bonding with Lys residues of cytochrome c than a single Asp residue (Shalaeva et al., 2015). By analogy, in case of the tau protein, it is tempting to speculate that several bystanding phosphate residues in higher vertebrates could sustain stronger bonding with the physiological partners—Arg or Lys residues—than a single phosphate group. It was shown that the strength of a bidentate bonding of an Arg residue even with a single phosphate group is compatible with that of a covalent bond (Woods and Ferré, 2005). A bifurcated or bidentate binding between clustered phosphate groups of tau and Arg/Lys residues of its physiological partner(s) could yield an even more tight interaction and, in addition, provide more control possibilities by constraining the mobility of residues involved.

Taken together our data provide evidence that novel, non-MT related tau interactions developed during evolution, which are mediated by tau’s N-terminal projection region (NTR and PRR). These evolutionary novel interactions relate to regulation of localization to membranes and regulation of apoptosis, i.e., biological processes, which may be of pathological relevance with respect to tau localization and cell survival. Furthermore, the data indicate that the number of predicted phosphorylation sites in the PRR showed a strong increase with divergence time and show that novel clusters of phosphorylatable residues developed during evolution in the PRR and in an alternatively spliced exon in the NTR. Thus, the data suggest that the propensity of regulation of tau function by phosphorylation (and potential miss-regulation during disease) developed during evolution and may point to isoform-specific regulatory mechanisms of tau isoforms containing N-terminal inserts. In view of the “antagonistic pleiotropy hypothesis,” an increase in phosphorylatable residues in the PRR during evolution could be beneficial for the organism’s fitness by providing increased ability for regulated interactions, while it would be detrimental at higher age due to increased propensity for miss-regulation and aggregation.

## Data Availability

All datasets generated for this study are included in the manuscript and/or the Supplementary Files.

## Author Contributions

NT, AM and RB designed the research. NT performed the research. NT, LB, AM and RB analyzed, interpreted the data and wrote the article.

## Conflict of Interest Statement

The authors declare that the research was conducted in the absence of any commercial or financial relationships that could be construed as a potential conflict of interest.

## Footnotes

^1^uniprot.org, release 04/2019

^2^ebi.ac.uk/QuickGO

^3^pymol.org

^4^ncbi.nlm.nih.gov/refseq

^5^timetree.org

^6^phosphosite.org/

^7^cbs.dtu.dk/services/NetPhos/

## Supplementary Material

FIGURE S1 Disorder prediction from different algorithms. The average of slope values from all selected algorithms for full-length tau and all selected regions (NTR, PRR, MBR and CTR) are shown. Regions are color-coded as shown in Figure 2. Note, that NTR and PRR mainly show an increase in disorder, while the disorder of the CTR decreased.

FIGURE S2 Comparison of average predicted disorder of tau sequences and its parts for the most represented taxons (i.e., bony fish, birds and mammals, color-coded from yellow to orange). **(A)** Boxplots for all selected disorder prediction algorithms are shown. The background color corresponds to regions of tau as shown in Figure 2. Statistical significance was derived from Mann–Whitney test. **p* < 0.05, ***p* < 0.01, ****p* < 0.001. **(B)** Numbers of differentially changed disorder for tau sequences divided into regions in selected taxons is shown. Here, table for one level of significance is shown, *p*-value < 0.05. Note that MBR shows a continuous increase in disorder (MBR), NTR and full-length tau mostly show increase from fish to birds and from fish to mammals, however, in some cases a decrease in disorder from birds to mammals was prominent and finally, CTR shows a continuous decrease in predicted disorder.

FIGURE S3 Comparison of numbers of predicted phosphorylation sites of tau and its regions for most represented taxons (i.e., bony fish, birds and mammals; color-coded from yellow to orange). **(A)** Boxplots for phosphorylation of tau and its regions are shown. The background colors and filling colors of boxplot correspond to the residue that was predicted to be phosphorylated (all sites—light gray, serine residues—turquoise, threonine residues—dark yellow and tyrosine residues—magenta). Statistical significance was derived from Mann–Whitney U test. **p* < 0.05, ***p* < 0.01, ****p* < 0.001. **(B)** Comparison of numbers of predicted phosphorylation sites of tau sequences and its parts for most represented taxons (i.e., bony fish, birds and mammals). Numbers of differentially changed disorder for tau sequences divided into regions in selected taxons is shown. Here, table for one level of significance is shown, *p*-value < 0.05.

## Abbreviations

aa: amino acid
AD: Alzheimer’s disease
BP: biological process
CNS: central nervous system
CTR: carboxy-terminal region
GO: Gene Ontology
IDP: intrinsically disordered protein
IDR: intrinsically disordered region
MAP: microtubule-associated protein
MBR: microtubule-binding region
MCI: mild cognitive impairment
MT: microtubule
NFT: neurofibrillary tangle
NTR: amino-terminal region
PHF: paired helical filament
PKA: cAMP-dependent protein kinase
PRR: proline-rich region
PTM: posttranslational modification.
