# Supplementary figures and images for: The Evolution of Tau Phosphorylation and Interactions
Source: Front Aging Neurosci. 2019 Sep 18;11:256. doi: 10.3389/fnagi.2019.00256 (PMC6759874; doi:10.3389/fnagi.2019.00256)

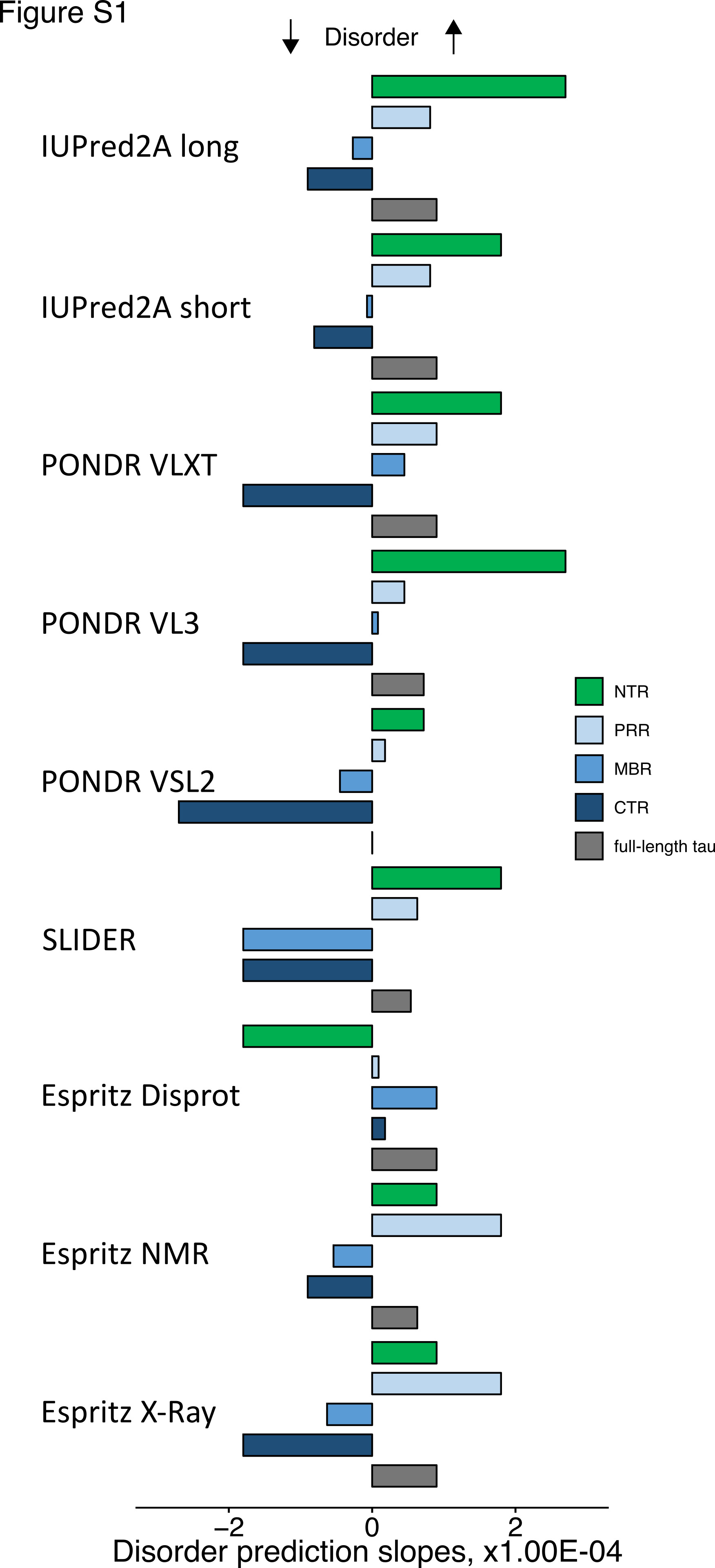

Supplement: FIGURE S1 — Disorder prediction from different algorithms. The average of slope values from all selected algorithms for full-length tau and all selected regions (NTR, PRR, MBR and CTR) are shown. Regions are color-coded as shown in Figure 2. Note, that NTR and PRR mainly show an increase in disorder, while the disorder of the CTR decreased. [file Image_1.JPEG]

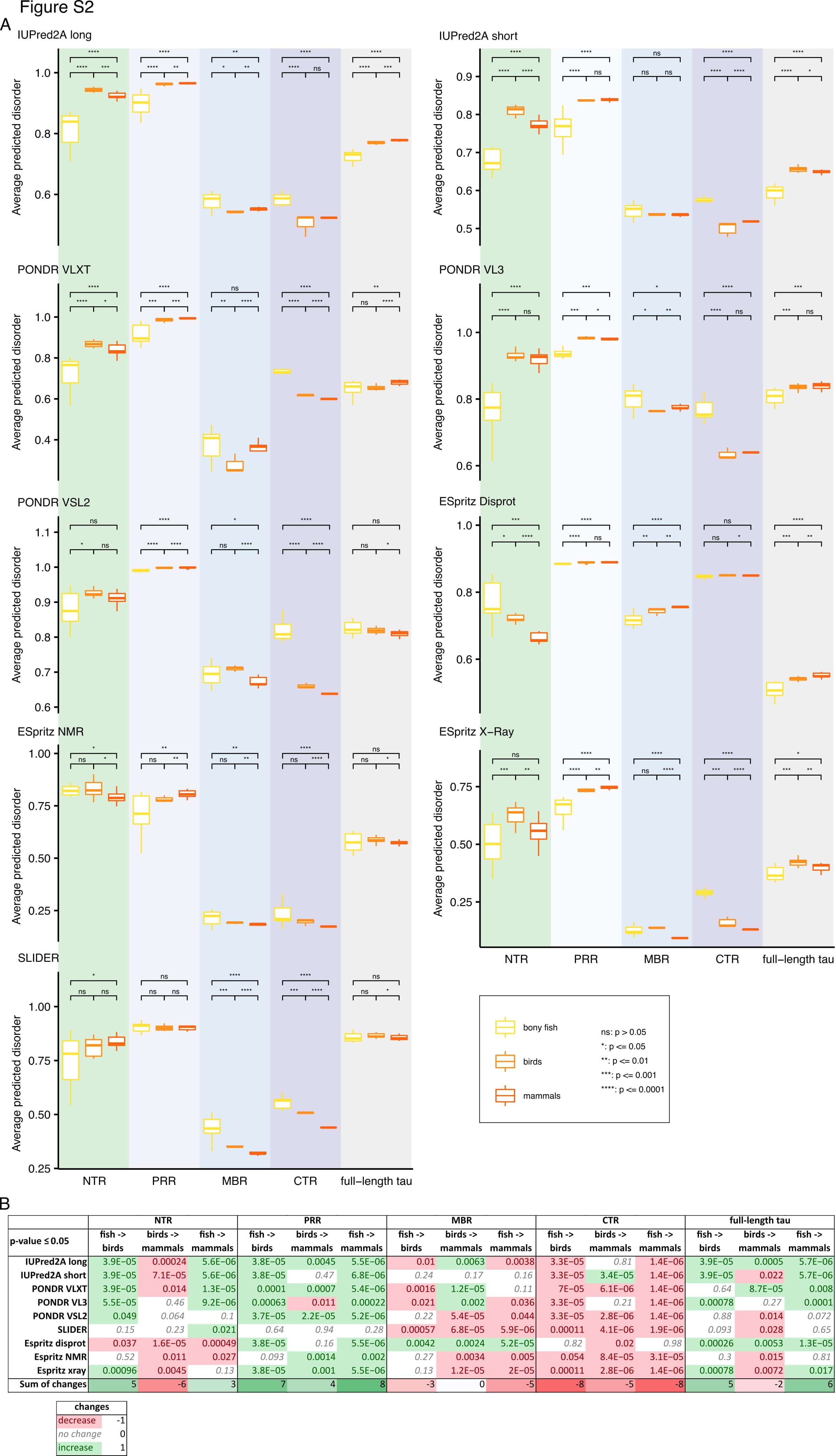

Supplement: FIGURE S2 — Comparison of average predicted disorder of tau sequences and its parts for the most represented taxons (i.e., bony fish, birds and mammals, color-coded from yellow to orange). (A) Boxplots for all selected disorder prediction algorithms are shown. The background color corresponds to regions of tau as shown in Figure 2. Statistical significance was derived from Mann–Whitney test. *p < 0.05, **p < 0.01, ***p < 0.001. (B) Numbers of differentially changed disorder for tau sequences divided into regions in selected taxons is shown. Here, table for one level of significance is shown, p-value < 0.05. Note that MBR shows a continuous increase in disorder (MBR), NTR and full-length tau mostly show increase from fish to birds and from fish to mammals, however, in some cases a decrease in disorder from birds to mammals was prominent and finally, CTR shows a continuous decrease in predicted disorder. [file Image_2.JPEG]

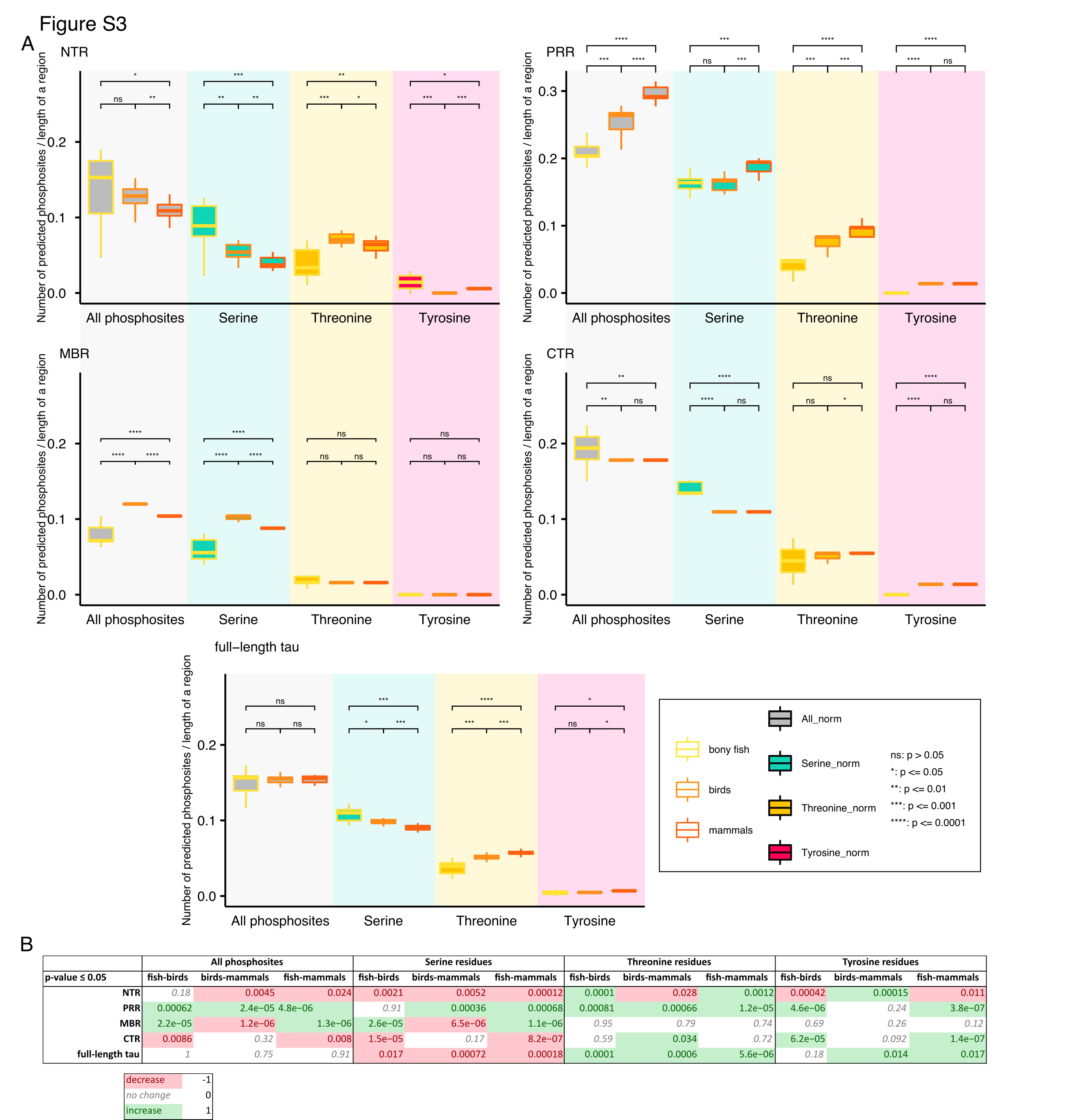

Supplement: FIGURE S3 — Comparison of numbers of predicted phosphorylation sites of tau and its regions for most represented taxons (i.e., bony fish, birds and mammals; color-coded from yellow to orange). (A) Boxplots for phosphorylation of tau and its regions are shown. The background colors and filling colors of boxplot correspond to the residue that was predicted to be phosphorylated (all sites—light gray, serine residues—turquoise, threonine residues—dark yellow and tyrosine residues—magenta). Statistical significance was derived from Mann–Whitney U test. *p < 0.05, **p < 0.01, ***p < 0.001. (B) Comparison of numbers of predicted phosphorylation sites of tau sequences and its parts for most represented taxons (i.e., bony fish, birds and mammals). Numbers of differentially changed disorder for tau sequences divided into regions in selected taxons is shown. Here, table for one level of significance is shown, p-value < 0.05. [file Image_3.jpg]
