# Supplementary material for: The Evolution of Tau Phosphorylation and Interactions
Source: Front Aging Neurosci. 2019 Sep 18;11:256. doi: 10.3389/fnagi.2019.00256 (PMC6759874; doi:10.3389/fnagi.2019.00256)
Supplement: Supplementary file 4 [file Table_1.DOCX]

**Table S1.** Genes and Uniprot IDs for human genes coding for interaction partners from Table 1.

| **N-terminal projection region (NTR)** | | **Proline-rich region (PRR)** | | **Microtubule-binding region (MBR) and C-terminal region (CTR)** | | **Additional interactions (not mapped)** | |
| --- | --- | --- | --- | --- | --- | --- | --- |
| **Genes** | **Uniprot IDs** | **Genes** | **Uniprot IDs** | **Genes** | **Uniprot IDs** | **Genes** | **Uniprot IDs** |
| MAPRE1 | Q15691 | BIN1 | O00499 | HSPA8 | P11142 | SNCA | P37840 |
| MAPRE3 | Q9UPY8 | SFN | P31947 | HSPB1 | P04792 | DDX | P26196 |
| ANXA2 | P07355 | PPP2R2A | P63151 | MAPRE1 | Q15691 | HSP90AB1 | P08238 |
| ANXA6 | P08133 | FYN | P06241 | MAPRE3 | Q9UPY8 | VDAC1 | P21796 |
| ANXA5 | P08758 | PIN1 | Q13526 | SFN | P31947 | TF | P02787 |
| YWHAB | P31946 | SRC | P12931 | APP | P05067 | FTH1 | P02794 |
| YWHAH | Q04917 |  |  | P4HB | P07237 | FKBP4 | Q02790 |
| SYN1 | P17600 |  |  | PPP2CA | P67775 | TIA1 | P31483 |
| SYT1 | P21579 |  |  | HDAC6 | Q9UBN7 | DAPK1 | P53355 |
| YWHAE | P62258 |  |  | PRNP | P04156 | LRRK2 | Q5S007 |
| YWHAG | P61981 |  |  | PPP2CA | P67775 | CLU | P10909 |
| YWHAZ | P63104 |  |  | MAPT | P10636 | YWHAZ | P63104 |
| SFN | P31947 |  |  | ACTB | P60709 | DNM1L | O00429 |
| VAMP2 | P63027 |  |  | CALM1 | P0DP23 | SYN1 | P17600 |
| PRNP | P04156 |  |  |  |  | SYT1 | P21579 |
| DCTN1 | Q14203 |  |  |  |  | VAMP2 | P63027 |
| GSK3B | P49841 |  |  |  |  | MAPK8IP1 | Q9UQF2 |
| ACTB | P60709 |  |  |  |  | APBB1 | O00213 |
|  |  |  |  |  |  | AATF | Q9NY61 |
|  |  |  |  |  |  | S100B | P04271 |
|  |  |  |  |  |  | CDK2 | P24941 |
|  |  |  |  |  |  | PSEN1 | P49768 |
|  |  |  |  |  |  | APOE | P02649 |
|  |  |  |  |  |  | SPTAN1 | Q13813 |
